# An EZ-Diffusion Model Analysis of Attentional Ability in Patients With Retinal Pigmentosa

**DOI:** 10.3389/fnins.2020.583493

**Published:** 2021-01-11

**Authors:** Yan-Lin Luo, Yuan-Ying Wang, Su-Fang Zhu, Li Zhao, Yan-Ling Yin, Meng-Wen Geng, Chu-Qi Lei, Yan-Hui Yang, Jun-Fa Li, Guo-Xin Ni

**Affiliations:** ^1^Department of Neurobiology, Capital Medical University, Beijing, China; ^2^Second Hospital of Armed Police Beijing Office, Beijing, China; ^3^Xuanwu Hospital, Capital Medical University, Beijing, China; ^4^School of Sports Medicine and Rehabilitation, Beijing Sport University, Beijing, China

**Keywords:** retinal pigmentosa, EZ-diffusion model, attentional orientation, attentional flexibility, attentional inhibition

## Abstract

Retinitis pigmentosa (RP) is characterized by visual acuity decrease and visual field loss. However, the impact of visual field loss on the cognitive performance of RP patients remains unknown. In the present study, in order to understand whether and how RP affects spatial processing and attentional function, one spatial processing task and three attentional tasks were conducted on RP patients and healthy controls. In addition, an EZ-diffusion model was performed for further data analysis with four parameters, mean decision time, non-decision time, drift rate, and boundary separation. It was found that in the spatial processing task, compared with the control group, the RP group exhibited a slower response speed in large and medium visual eccentricities, and slower drift rate for the large stimulus, which is strongly verified by the significant linear correlation between the visual field eccentricity with both reaction time (*p* = 0.047) and non-decision time (*p* = 0.043) in RP patients. In the attentional orienting task and the attentional switching task, RP exerted a reduction of speed and an increase of non-decision time on every condition, with a decrease of drift rate in the orienting task and boundary separation in the switching task. In addition, the switching cost for large stimulus was observed in the control group but not in the RP group. The stop-signal task demonstrated similar inhibition function between the two groups. These findings implied that RP exerted the impairment of spatial cognition correlated with the visual field eccentricity, mainly in the peripheral visual field. Moreover, specific to the peripheral visual field, RP patients had deficits in the attentional orienting and flexibility but not in the attentional inhibition.

## Introduction

Retinitis pigmentosa (RP) is a group of hereditary retinal diseases characterized functionally by the degeneration of rod and cone photoreceptors (Hamel, 2006). Progressive peripheral visual field loss, also known as visual field constriction or tunnel vision, is one of the most significant clinical manifestations (Gordon and Johns, 1984).

Visual dysfunction has a negative impact in the information processing (Turatto et al., 1999). Specifically, RP leads to a deficit in visual information perception and motor perception. For example, RP patients experience the compression of spatial information and perceptual magnification in their visual fields (Wittich et al., 2011), a phenomenon known as perceptual filling-out (Temme et al., 1985). Besides, RP patients exhibit an increase in the minimum motion threshold and a decrease in the maximum motion threshold in motion coherence (Alexander et al., 1998). It is also suggested that RP patients with severe vision loss failed to perceive changes in various dot contrasts and sizes, implying that information processing is closely related to the remnant vision (Alexander et al., 1999).

Although accumulating evidence suggests a general impairment in visual information processing in RP patients (Herse, 2005; Wittich et al., 2011), to date, no precise quantitative account exists of their special cognitive performance induced by visual field loss, especially the attentional ability which is fundamental for cognitive processing in the peripheral and central visual fields. One way to quantify the cognitive function is by conducting an observational experiment to obtain such information as reaction time (RT) and accuracy on a specific experiment. Actually, people tend to slow down response speed for a higher accuracy (Ratcliff et al., 2016; Wagenmakers et al., 2017). In order to propose concrete mechanisms that drive observed behavior and explore the underlying processes that determine performance on an experiment, the effect of such speed-accuracy trade-off should be taken into consideration during the process of data analysis. As successful cognitive process models, the drift diffusion model (Ratcliff, 1978; Ratcliff et al., 2016) and its simplified version EZ-diffusion model (Moustafa et al., 2015) have been widely applied to compensate for the insensitivity of the original data of accuracy and RT, and the insufficient consideration of the speed-accuracy trade-off.

In this study, four experiments were established to investigate the cognitive impairment in the central and peripheral visual fields of RP patients. Experiment 1 aimed to evaluate the spatial processing in the periphery and central visual fields with various field sizes. Due to RP-related progressive loss in the peripheral visual field, the following experiments focused specifically on the peripheral visual field. Accordingly, the ability of basic attentional orienting was examined in Experiment 2, and the attentional inhibition and attentional flexibility were tested in Experiments 3 and 4, respectively. In the last part of our study, we analyzed the data with the EZ-diffusion model and tried to explore the duration of spatial and attentional processes including the decision and non-decision time in the central and peripheral visual fields.

## Materials and Methods

### Patients and Clinical Examinations

We have no prior beliefs or pilot data to estimate the minimum required sample size to observe a significant difference between groups. We recruited 19 RP patients and 13 healthy subjects for Experiment 1 because this number reflects the average sample size in similar RP studies (Alexander et al., 1998, 1999; Wittich et al., 2011). Based on the results of Experiment 1, we conducted a power analysis by using an alpha of 0.05, one-tailed, power of 0.8, and the effect size from Experiment 1. The effect size was computed to reflect a between-subject design. We found a minimum required sample size to be 7 for RP patients and 5 for healthy subjects. Thus, same number of participants were enrolled for each experiment.

In this study, RP patients (RP group) were recruited from the Second Hospital of Beijing Armed Police Corps office. Healthy subjects chosen from the patients’ family and matched by age and gender serve as the control group. For each subject, the following examinations were taken, including E decimal charts, visual field evaluations, slit-lamp biomicroscopy, and fundus inspection. The research protocol was approved by the Ethics Committee of the Capital Medical University, China. The complete details of the entire study design and procedures involved were in accordance with the Declaration of Helsinki. Written informed consent was obtained prior to participation.

RP patients were included only if they fulfilled the following criteria: (1) were ≥ 18 years of age; (2) with night blindness; (3) with peripheral visual loss; (4) with typical abnormal fundus appearance, including change in retinal pigmented epithelium; (5) without movement disorders;, and (6) without achromatopsia. Healthy subjects should have either normal or corrected normal vision acuity (≥ 1.0), clear ocular media, and normal-appearing fundi. For both groups, exclusion criteria included a history of major physiological and psychological diseases.

### Materials, Apparatus, and Procedures

**FIGURE 1:**
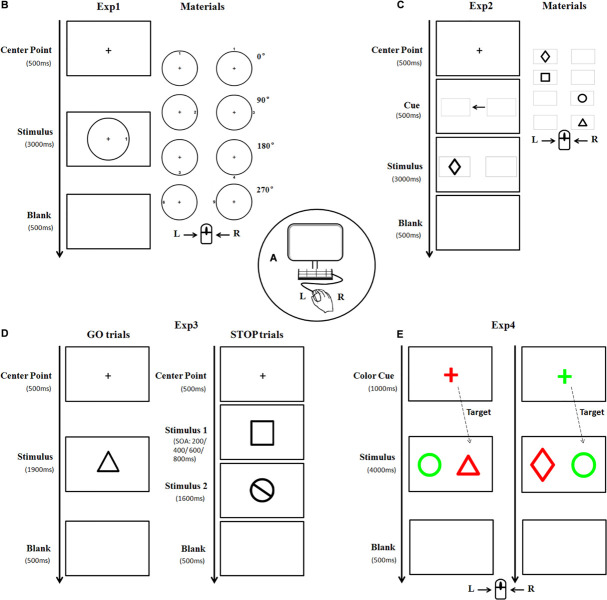
A diagram illustrating the experimental materials and procedures. Subjects were seated in front of a computer screen **(A)** to perform four experiments in a random order. In Experiment 1, the stimulus picture consisted of one circle and one target number. The target number was oriented clockwise in 0, 90, 180, or 270° either inside or outside the circle, respectively **(B)**. Each subject was asked to press the left/right key of the mouse according to the outer/inner location of the targets. Stimulus in Experiment 2 was presented as one of four black geometrical figures **(C)**. Each subject pressed the key according to the left/right location of the target. Experiment 3 consisted of GO and STOP trials. The materials in the GO trial were similar to that in Experiment 2, whereas, the stimulus in the STOP trial is a black-stop symbol **(D)**. Subjects were instructed to press the left key of the mouse in the absence of STOP stimulus, otherwise, withhold the action. As for Experiment 4, the stimulus was a pair of different geometric figures (as described in Experiment 2) placed horizontally, with one highlighted in *red* and the other in *green* **(E)**. The figure with the same color as the cue was regarded as the target. Subjects were asked to press the left/right key of the mouse if the target is (or is not) a triangle figure (Exp, experiment).

In this study, each participant completed four experiments in a random order in a soundproof, light-isolated chamber with a constant temperature of 25°C (SD = 1°C) (Figure 1). The participant was seated in front of a 17-in. computer screen (Lenovo ColorSync), positioned approximately 57 cm distant from his eyes. Stimulus presentation was controlled with E-Prime (Psychology Software Tools Inc., Pittsburgh, United States). For each experiment, training trial identical to the formal one was initially conducted 20 times.

Experiment 1 (The spatial processing task): The stimulus picture consisted of one black circle and one black target number. There were three sizes of circle with a visual angle of 3.5°, 5°, and 7°, respectively, horizontally from the center fixation. The target number (between 1 and 9) was oriented clockwise in 0°, 90°, 180°, or 270° either inside or outside the circle, with 0° defined as the upright of the circle. The font size of the number was either 22-px, 31-px, or 44-px, congruent with the size of the circle. There was a total of 132 trials with stimulus of each size appearing randomly and equally. The formal trial began with the appearance of a central fixation cross “+” for 500 ms, followed by a stimulus for 3,000 ms. Each participant was instructed to press the left (or right) key of the mouse at the appearance of target number inside (or outside) the circle. Once the button was pressed, the stimulus would disappear followed by a 500 ms blank interval, and the next trial began (Figure 1B).

Experiment 2 (The attentional orienting task): The target was one of four black geometrical figures (rectangle, diamond, circle, and triangle), presented with 7° in eccentricity horizontally from the center fixation. There were a total of 146 trials with figure form and location appeared randomly and equally. The formal trial began with the appearance of a central fixation cross “+” for 500 ms, followed by two identical gray horizontal boxes (width = 7.4°, *SD* = 0°; height = 4.7°, *SD* = 0°) presented for 500 ms with a black central arrow in between. Afterward, a target appeared inside either the ipsilateral (96, 65.8%) or contralateral (50, 34.2%) box for 3,000 ms (Figure 1C). Each participant was instructed to quickly press the left (or right) key of the mouse according to the location (left or right) of the box where the target figure appeared.

Experiment 3 (The stop-signal task): This experiment consisted of GO and STOP trials. Similar to Experiment 2, the stimulus in GO trial (53.8%) was one of four black geometrical figures, whereas, the stimulus in STOP trial (46.2%) was a black-stop symbol. These stimuli appeared in either large size or small size (width = 14°/10°, *SD* = 0°; height = 14.13°/11.1°, **SD** = 2.40°/1.33°, presented at 7 and 5° in eccentricity horizontally from the center fixation, respectively). After the appearance of a central fixation cross “+” for 500 ms, a GO stimulus would appear for 1,900 ms, sometimes followed by a STOP stimulus with stimulus onset asynchronies (SOAs) of either 200, 400, 600, or 800 ms. Each participant was instructed to press the left key of mouse in the absence of STOP stimulus, otherwise, withhold his action (Figure 1D). There were a total of 208 trials in this experiment, including 112 GO trials and 96 STOP trials (24 STOP trials for each SOA).

Experiment 4 (The attentional switching task): Stimuli in this experiment were a pair of horizontally placed geometric figures (as described in Experiment 2), highlighted in red (RGB: 255, 0, 0) and green (RGB: 0, 255, 0), respectively. There was a total of 186 trials with large stimulus (width = 5°, SD = 0°; height = 5.57°, SD = 1.32°) and small stimulus (width = 3.5°, SD = 0°; height = 3.88°, SD = 0.85°), which were presented at 10 and 7° in eccentricity horizontally from the center fixation, respectively, and appeared randomly and equally. A colorful central fixation cross “+” (font size of 64-px) was first displayed in either red (RGB: 255, 0, 0) or green (RGB: 0, 255, 0) randomly as a cue for 1,000 ms, followed by the appearance of a stimulus for 3,000 ms. The figure with the same color of the cue was regarded as the target. Each participant was instructed to press the left (or right) key of the mouse if the target was (or was not) a triangle figure (Figure 1E).

### The EZ-Diffusion Model

Drift diffusion model and its simplified version EZ-diffusion model have been widely applied in various cognitive tasks, including the direction discrimination task (Metin et al., 2013), reward and punishment learning task (Moustafa et al., 2015), response signal and Go/No-Go tasks, value-based decision making, as well as conflict tasks for ADHD (Metin et al., 2013), schizophrenia (Moustafa et al., 2015), and healthy groups (Ratcliff et al., 2016). In EZ-diffusion model, the observed RT can be separated into mean decision time (MDT) and non-decision time (Ter). Ter can be further divided into information encoding time (the period before information accumulation to a response) and motor time (the period after information accumulation) (Wagenmakers et al., 2017). Information accumulation begins at a certain level, proceeds over time with drift rate [information processing speed (*v*)], and halts once either the upper or the lower boundary is reached, during which the distance is called boundary separation (A).

### Data Analysis and Statistical Analysis

Accuracy (ACC) was defined as the number of correct response (in percentage) in relation to the original response number, and RT as the time period between the stimulus onset and the participant’s response. Only trials with RT in the range of 200 and 3,000 ms were included for further data analysis (98.62% of all correct trials). However, trials with appropriate RT, but the incorrect response would be excluded from the analysis of RT. In addition, several EZ-diffusion model parameters were calculated according to the formula described elsewhere, including *v*, *A*, *MDT*, and *Ter* (Wagenmakers et al., 2017).

Prior to statistical analyses, we compared the expected data with the observed data by coefficients analysis using R software to find whether this model fits our data quite well or the statistical analysis was valid enough. The following results indicated *R*^2^ = 0.125 and 0.222 for accuracy and RT in Experiment 1, *R*^2^ = 0.105 and 0.242 in Experiment 2, and *R*^2^ = 0.147 and 0.149 in Experiment 4, respectively, which verified that the EZ model provides a good fit to our data and can confidently investigate the differences in performance between RP patients and controls.

Statistical analyses were performed by using SigmaStat 3.5 (SAS Institute Inc., Cary, NC). Group differences in demographics, clinical data, and experimental data were analyzed using independent samples *t*-tests (for quantitative data), Fisher’s exact test (for categorical data), and two-way ANOVA (for quantitative data) where appropriate. Holm-Sidak test was conducted as *post hoc* test to analyze differences between and within subjects. The correlation between the RT, EZ-diffusion model parameters, and the visual field eccentricity was processed using Pearson correlation in Experiment 1. All tests were two sided, and statistical significance was defined as *p* < 0.05 for multiple comparisons.

## Results

### Demographics

Table 1 presents the demographic and clinical characteristics of the study population. There was no significant difference between the two groups in terms of sex ratio, age, educational attainment, Edinburgh hand scale, and Mini-Mental State Examination (MMSE), respectively. However, in either right or left eye, a significant lower corrected visual acuity was found in the RP group (right eye: 95% CI, 0.27–0.42; *p* < 0.001; left eye: 95% CI, 0.13–0.25; *p* < 0.001), when compared with the control group (right eye: 95% CI, 0.99–1.08; left eye: 95% CI, 0.98–1.04). In this study, RP patients had disease duration of 95% CI, 5.31–9.63 years, among which 9 patients had a family history (Table 1).

**TABLE 1:** Demographics and clinical data in RP and control groups.

	**RP group (95% CI)**	**Control group (95% CI)**	***T*−value**	***P*-value (*t*-test)**
**95% CI**				
Subject number	19	13		
Male/Female	12/7	9/4		1.000^#^
Age (years old)	34 (29.15–38.94)	28.28 (26.02–30.47)	1.905	0.066
Education levels (years)	12.63 (10.48–14.78)	15.23 (14.33–16.13)	–2.599	0.053
Edinburgh hand scale	87.91 (82.25–93.55)	87.89 (79.47–96.31)	0.003	0.998
MMSE	27.94 (27.45–28.44)	28.00 (27.01–28.98)	–0.098	0.923
**VA (log MAR)**				
RE (mean ± SD)	0.34 (0.27–0.42)	1.04 (0.99–1.08)	19.275	< 0.001
LE (mean ± SD)	0.19 (0.13–0.25)	1.02 (0.98–1.04)	15.762	< 0.001
Family history (*n*, %)	9 (47%)			
Disease duration (years)	7.47 (5.31–9.63)			

*RP, retinal pigmentosa; MMSE, Mini-Mental State Examination; VA, visual acuity; MAR, minimum angle of resolution; RE, right eye; LE, left eye. ^#^Fisher’s exact test.*

### Effect of RP on Spatial Processing

**FIGURE 2:**
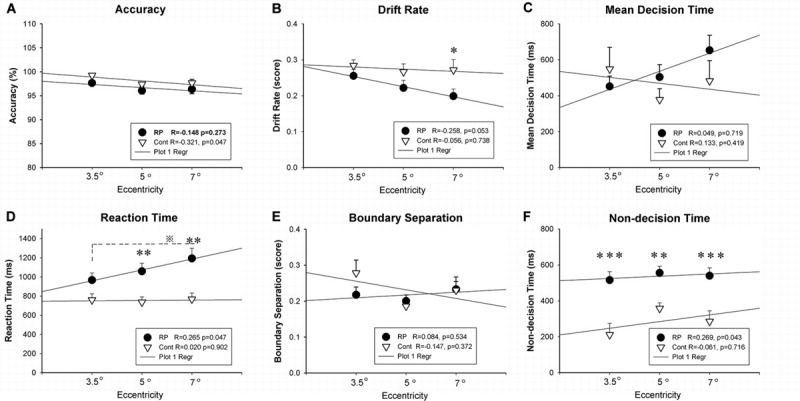
The results for the spatial processing task (Experiment 1). Results of accuracy **(A)**, RT **(D)**, and EZ-diffusion model parameters, as well as their correlations with visual field eccentricity were presented in two groups. The RP group responded much slower to large (eccentricity 7°) and medium stimuli (eccentricity 5°) when compared with the control group. In addition, the RP group presented significantly smaller *v* for the large stimulus **(B)** and longer *Ter* at each eccentricity than the control group **(F)**, respectively. There were no significant differences between the two groups in mean decision time **(C)** and boundary separation **(E)** at each eccentricity, respectively. In addition, a significant correlation of visual field eccentricity was observed with RT **(D)** and Ter **(F)** in the RP group, respectively. (RP, retinal pigmentosa; Cont, control). ^∗^*p* < 0.05, ^∗∗^*p* < 0.01, ^∗∗∗^*p* < 0.001, RP group vs. control group; ^※^*p* < 0.05, eccentricity 7° vs. eccentricity 5°.

Experiment 1 was designed to evaluate the spatial processing in the peripheral and central visual fields with various field sizes. Figure 2 showed the results of ACC, RT, and EZ-diffusion model parameters in two groups. A distinct longer RT was observed in the RP group in comparison with the control group for large (eccentricity 7°) (RP: 95% CI, 970.51–1,416.02 ms; control: 95% CI, 626.03–909.45 ms; *p* = 0.001) and medium stimuli (eccentricity 5°) (RP: 95% CI, 881.97–1,236.94 ms; control: 95% CI, 881.97–1,236.94 ms; *p* = 0.008), respectively. While there was no significant change of RT for the small stimulus (eccentricity 3.5°) between two groups (RP: 95% CI, 811.45–1,123.46 ms; control, 95% CI, 619.30–899.95 ms), statistically significant difference between large and small stimuli was observed in the RP group (*p* = 0.039), suggesting an impaired spatial processing in RP patients in the peripheral visual field.

For the analysis on the EZ-diffusion model, a significantly smaller *v* was revealed in the RP group only in a large field (95% CI, 0.16–0.24; *p* = 0.020) when compared with the control group (95% CI, 0.21–0.33). This result indicated a slower processing speed in RP patients in the peripheral visual field. Moreover, the deficit in information coding was also observed in RP patients, which was proved by the *Ter* significantly longer than their healthy counterparts in either large (RP: 95% CI, 448.26–632.26 ms; control, 95% CI, 156.74–414.31 ms; *p* < 0.001), medium (RP: 95% CI, 477.77–633.90 ms; control, 95% CI, 289.94–426.16 ms; *p* = 0.005), or small visual field (RP: 95% CI, 416.87–614.63 ms; control, 95% CI, 71.22–351.43 ms; *p* < 0.001). Taken together, RP patients presented a far more serious defect phenomenon on the spatial processing in their peripheral visual field, which may relate to the peripheral visual field loss in these patients (Figure 2). Additionally, by Pearson correlation analysis, significant correlation of visual field eccentricity was observed with RT (*R* = 0.265, *p* = 0.047) and Ter (*R* = 0.269, *p* = 0.0430) in RP patients, respectively. Whereas no significant correlation of visual field eccentricity was found with other EZ-diffusion model parameters in RP patients, as well as RT and all EZ-diffusion model parameters in the control group (Figure 2).

### Effect of RP on the Attentional Orienting

**FIGURE 3:**
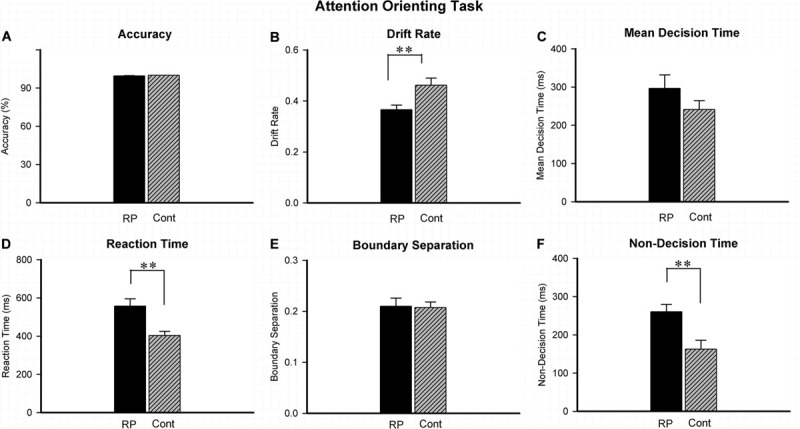
The results for the attentional orienting task (Experiment 2). Results of accuracy **(A)**, RT **(D)**, and EZ-diffusion model parameters were presented in two groups. Compared with the control group, the RP group responded much slower in attentional orientation. In addition, the RP group presented significantly smaller *v* **(B)** and longer Ter than the control group **(F)**, respectively. There were no significant differences between the two groups in mean decision time **(C)** and boundary separation **(E)**, respectively. (RP, retinal pigmentosa; Cont, control). ^∗∗^*p* < 0.01, RP group vs. control group.

Experiment 2 was conducted to investigate the attentional orienting by judging the location of stimulus in the peripheral visual field. The results of ACC, RT, and several EZ-diffusion model parameters are presented in Figure 3. The effect of attentional orienting can be reflected by comparing the high proportion of valid stimuli (ratio > 70%) with the low proportion of invalid stimuli (ratio < 30%). However, in this study, no significant effect was found in RT data of 96 valid stimuli (ipsilateral, 65.8%) in comparison with 50 invalid stimuli (contralateral, 34.2%), probably because the valid/invalid ratio was not particularly high. Therefore, all the data (both the valid and invalid stimuli) were finally integrated to analyze the difference between the RP and the control groups. Compared with their healthy counterparts (95% CI, 356.56–450.97 ms), RP patients exhibited a much longer RT (95% CI, 477.12–636.83 ms; *p* = 0.004). Under the EZ-diffusion model analysis, there was no statistical significance of group between difference in neither *A* nor *MDT*. However, the RP group exhibited a distinct smaller *v* (95% CI, 0.33–0.40; *p* = 0.006) and longer Ter (95% CI, 219.39–301.24 ms; *p* = 0.003) than the control group (*v:* 95% CI, 0.46–0.52; Ter*:* 95% CI, 112.11–213.25 ms), indicating that RP could lead to a deficit in the information processing speed and encoding speed (Figure 3).

### Effect of RP on the Attentional Inhibition

**FIGURE 4:**
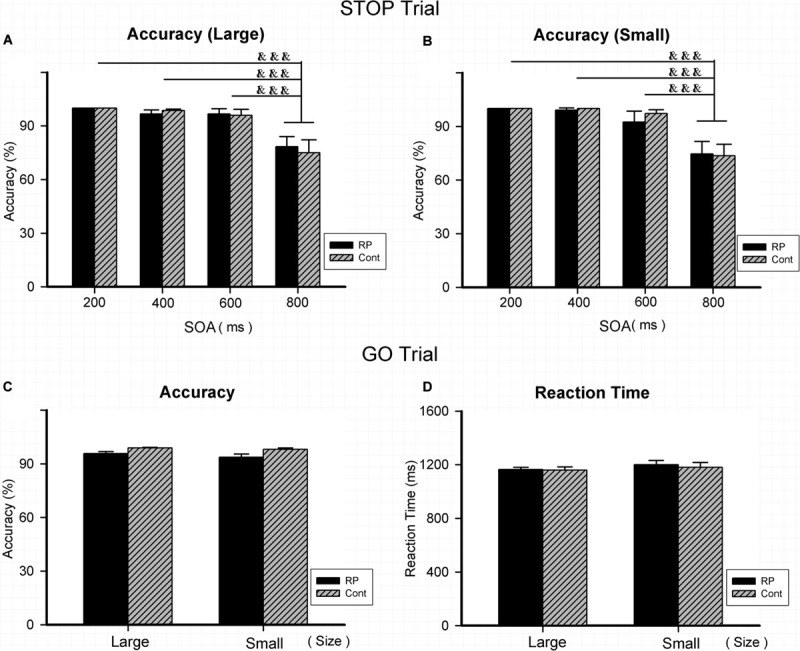
The results for the stop-signal task (Experiment 3). Results of accuracy and RT were presented for the STOP and GO trials in two groups. As for the STOP trials, there was no significant difference between the two groups in accuracy for either large **(A)** or small stimulus **(B)**. In addition, regardless of group and stimulus size, significantly lower ACC was found with 800 ms SOA, compared with that with 200, 400, and 600 ms SOA, respectively. As for the GO trials, statistically significant difference did not exist between the two groups for large and small stimuli in either accuracy **(C)** or RT **(D)**, respectively. (RP, retinal pigmentosa; Cont, control; SOAs, stimulus onset asynchronies). ^&&&^*p* < 0.001, compared with that with 800 ms.

Experiment 3 was conducted to assess the patient’s attentional inhibition by observing the ability of control in the peripheral visual field. Figure 4 illustrated the results of RT and ACC in two groups. In the GO and STOP trials, the statistically significant difference could not be evaluated between two groups in either ACC or RT. Results in GO trial indicated that RP patients had general performing ability, similar to their healthy counterparts. In addition, regardless of grouping and stimulus size, a significantly lower ACC was found with 800 ms SOA, compared with that with 200, 400, and 600 ms SOA, respectively. Results of ACC in the STOP trials suggested a similar outcome when treated with SOA in the two groups, indicating that the RP group presented similar symptom of the attentional inhibition when compared with the control group (Figure 4).

### Effect of RP on the Attentional Flexibility

**FIGURE 5:**
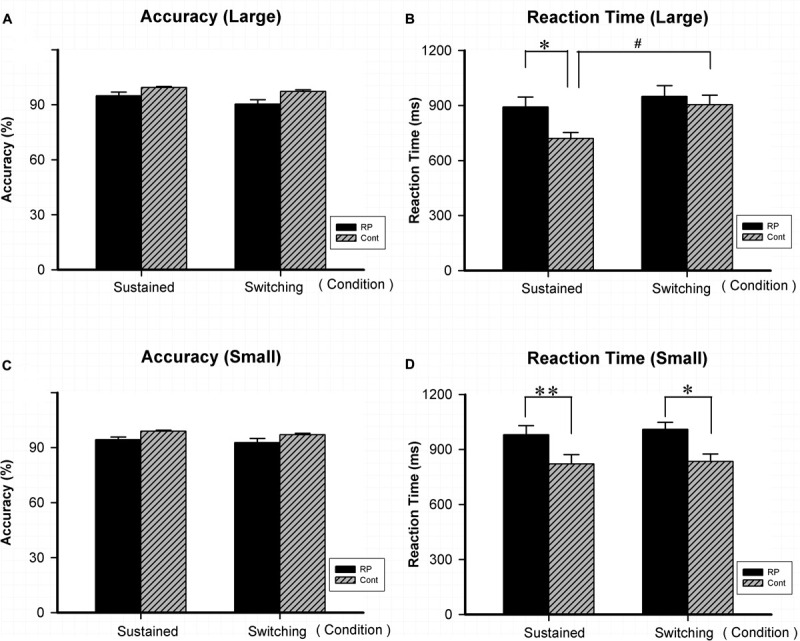
The results for the attentional switching task (Experiment 4). Results of accuracy and RT for large and small stimuli under sustained and switching conditions are presented. As for accuracy, there was no significant difference between the two groups under either sustained or switching condition for either large **(A)** or small stimulus **(C)**. As for RT, significantly worse performance was found in the RP group for small stimulus **(D)** under sustained and switching condition, as well as for large stimulus **(B)** under sustained condition. The switching cost (longer RT was found under switching condition than under sustained condition) for large stimulus existed in the control group, but *not* in the RP group. (RP, retinal pigmentosa; Cont, control). ^∗^*p* < 0.05, ^∗∗^*p* < 0.01, RP group vs. control group; ^#^*p* < 0.05, switching condition vs. sustained condition.

**FIGURE 6:**
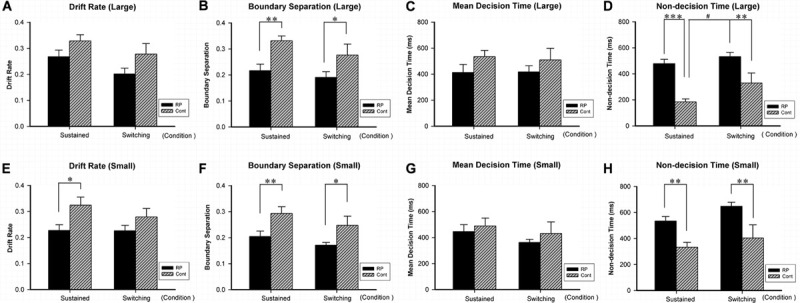
The results for the attentional switching task (Experiment 4). Results of EZ-diffusion model parameters for large **(A–D)** and small **(E–H)** stimuli under sustained and switching conditions are presented. Under sustained and switching conditions, significantly smaller *A* **(B,F)** and longer Ter **(D,H)** were observed in the RP group than the control group for both large and small stimuli, respectively. Besides, the RP group showed shorter Ter **(E)** for small stimulus under sustained condition. The switching cost (longer Ter was found under switching condition than under sustained condition) for large stimulus existed in the control group, but *not* in the RP group. (RP, retinal pigmentosa; Cont, control). ^∗^*p* < 0.05, ^∗∗^*p* < 0.01, ^∗∗∗^*p* < 0.001, RP group vs. control group; ^#^*p* < 0.05, switching condition vs. sustained condition.

As for Experiment 4, the attentional flexibility under sustained and switching conditions in the peripheral visual field was evaluated. Results of ACC, RT, and the EZ-diffusion model parameters in the two groups are presented in Figures 5, 6, respectively. The attentional flexibility is mainly reflected by the difference between the switching and the sustained conditions, which is the so-called switching cost. The greater the switching cost means the stronger the flexibility. Interestingly, the switching cost for large stimulus existing in the control group (sustained condition: 95% CI, 630.67–809.28 ms; switching condition: 95% CI, 763.65–1,044.71 ms; *p* = 0.044) was not observed in the RP group (sustained condition: 95% CI, 777.31–1005.89 ms; switching condition: 95% CI, 825.91–1,073.61 ms; *p* = 0.435) (Figure 5).

Significantly, RP patients presented a worse performance in RT when compared with their healthy counterparts for small stimulus under sustained (RP: 95% CI, 877.48–1,083.95 ms; control: 95% CI, 684.01–958.81 ms; *p* = 0.004) and switching condition (RP: 95% CI, 928.56–1,090.02 ms; control: 95% CI, 764.21–956.27 ms; *p* = 0.026), as well as for large stimulus under sustained condition (RP: 95% CI, 777.31–1,005.89 ms; control: 95% CI, 630.67–809.28 ms; *p* = 0.041). These findings suggested that RP led to the impairment in reaction speed under both conditions, with a more severe symptom under sustained condition.

By the EZ-diffusion model analysis, under sustained and switching conditions, significantly smaller *A* and longer Ter were observed in the RP group than the control group for both large and small stimuli, respectively (Figure 6). Besides, the RP group showed slower *v* for small stimulus under sustained condition (RP: 95% CI, 0.18–0.27; control: 95% CI, 0.26–0.39; *p* = 0.011). Notably, the switching cost for large stimulus existing in the control group in Ter (under sustained condition: 95% CI, 135.46–234.09 ms; under switching condition: 95% CI, 162.03–496.78 ms; *p* = 0.036) was not found in the RP group, which may be due to the considerably longer Ter in the RP group for large stimulus under sustained condition (RP: 95% CI, 408.15–548.95 ms; control: 95% CI, 135.46–234.09 ms; *p* < 0.001). Our findings in Ter explained the disappearance of switching cost in RT, since the RP group has longer encoding time than the control group under sustained condition (Figure 6).

## Discussion

The spatial processing and attentional ability of RP patients in both the central and peripheral visual fields has never been examined before, and we firstly reported the findings as mentioned in the “Result” section. Our findings demonstrated that RP patients exhibited impairment in spatial processing correlated with the visual field eccentricity and mainly in the peripheral visual field. Moreover, specific to the peripheral visual field, RP patients exhibited deficits in attentional orienting and flexibility, whereas no deficits were found in attentional inhibition.

Previously, the stimuli were presented at a visual angle of about 1, 3, or 5° horizontally from the central fixation point, and the deficits of spatial processing was only observed at the largest angle in RP patients (Wittich et al., 2011). Similar results were found in this work, in which the eccentricity range was expanded to 3.5, 5, and 7° from boundary to the center, respectively, and considerable impairment mainly existed at two bigger angles. EZ-diffusion model analysis further indicated that such slow performance in RP patients relates to the long non-decision time for each size stimulus and slow drift rate *v* for large stimulus. The similar phenomenon, also observed in myopic patients, was thought to be associated with the narrowed visual perception field at large eccentricity (Turatto et al., 1999). This could lead to the ignorance of patients on the peripheral visual stimulus and to allocate few attentional resources to the periphery field, causing a defective attentional orienting and processing (Turatto et al., 1999). In view of an even more severe visual field dysfunction than myopia patients, it is not surprisingly to find that RP patients, with progressive peripheral field loss, exhibited impairment of spatial processing in the peripheral field.

Our finding of significant correlation of visual field eccentricity with RT and Ter in RP patients provides additional evidence that the impairment of spatial processing speed was mainly caused by visual field loss. Nevertheless, many other factors should also be considered, including luminance contrast, spatial contrast, duration and receptor sampling density of stimuli, contrast sensitivity, glare sensitivity of human eye (Szlyk et al., 1995; Herse, 2005). It was suggested that RP patients may exhibit an elevation in threshold to vernier, letter, and grating visual acuity (Sandberg and Berson, 1983; Alexander et al., 1986, 1991, 1992a,b), a reduction in grating contrast sensitivity (Temme et al., 1985; Turano, 1991; Turano and Wang, 1992; Alexander et al., 1998), as well as a delay in flash detection and a loss of flicker sensitivity (Marmor, 1981; Alexander et al., 1995; Akeo et al., 2002). Notably, compared with their healthy counterparts, such influencing factors may have more severe impact on RP patients, and a slightly functional loss would be considerably amplified. For example, with the decrement of luminance, the environmental adaptability decreases and the contrast sensitivity threshold increases in RP patients, leading to their difficulty in walking, driving, reading the street signs, and crossing obstacles at night (Herse, 2005). In parallel, the slight changes of stimulation in pattern contrast (Michelson) or in temporal frequency sensitivity will cause more errors in the symmetry discrimination for RP patients, and the error number presents a functional change related to the visual field eccentricity (Szlyk et al., 1995). Due to the importance of the human eye characteristics, further investigations are warranted to understand the relationship among the spatial processing, visual field loss, and contrast sensitivity in RP patients.

As reported, an RP patient is prone to being tripped by obstacles during walking, implying an attentional deficit or orientation difficulty (Herse, 2005). Our findings from Experiment 2 suggested that there was a deficit in attentional orienting in the peripheral field (7° from the central fixation point). By the EZ-diffusion model analysis, the decrease of response speed in orienting was proved to be attributable to declined drift rate and increased non-decision time. It is widely recognized that attentional orienting is associated with the frontoparietal network, including the dorsal and ventral attentional network (Vincent et al., 2008; Farrant and Uddin, 2015). The attentional information processing through this network was reportedly delivered *via* primary visual area (V1), where the earliest neural activity of cognition was detected (Li, 1999; Chen et al., 2016). As demonstrated in myopic and strabismus amblyopia patients (Mori et al., 2002; Thiel and Sireteanu, 2009; Baranton et al., 2014), those with V1 lesion may be at risk of damaging the attentional network and therefore causing the orienting difficulty. In this regard, the decreased activities in V1, previously reported in RP patients (Ferreira et al., 2017) may at least partly interpret their attentional orienting deficit revealed in the present study.

Attentional switching refers to a process of reorganizing attentional set with the change of goal and task and therefore reflects the attentional flexibility. People usually keep the present attentional set active with sustained attention, and then activate a new one while leaving the previous one with switching attention (Mayr and Keele, 2000). Consequently, they tend to respond substantially slower and with higher error rate under switching condition than sustained condition (Monsell, 2003). Such discrepancy in RT between two kinds of trials is known as the attentional switching cost (Meiran and Chorev, 2005). It is worthwhile to notice that attentional switching cost only existed in large stimulus trial in the control group but not in either large or small stimulus trial in the RP group. The disappearance of the switching cost in RP patients should be ascribed to the lengthening of RT and information encoding time under sustained condition. RP patients presented a reduction of boundary separation and non-decision time under both sustained and switching conditions, especially under sustained condition. Such findings, taken together with the decrease in drift rate in the sustained trial with small size, implied that RP patients have difficulty in maintaining visual stability. An increase of eye movement was previously reported in RP patients during walking (Yoshida et al., 2014). Visual instability may relate to the narrowed visual field, since healthy subjects constricted to narrowed visual field presented increased pause frequency, prolongation of reading time, and increased eye movements during reading (Turano et al., 1993). The visual instability in RP patients could be explained by the insufficient sampling, the impaired spatial and temporal contrast sensitivity, the decreased threshold for motor perception, or the combination of the above (Wittich et al., 2011).

Several brain areas have already been reported in the process of sustained attention, such as prefrontal (Wilkins et al., 1987), parietal (Thakral and Slotnick, 2009), V1 area, and anterior cingulate cortex (Kerns et al., 2004; Silver and Ress, 2007). Among these areas, the first three were reported to be damaged in RP patients (Yoshida et al., 2014; Ferreira et al., 2017) which may result in the instability of sustained attention. Also, it is reported that activities of an attentional network including frontal and parietal areas were related to the drift rate in diffusion model (Karalunas et al., 2012), which is likely associated with the sustained attentional deficit in RP patients revealed in our study. Hence, further investigations are warranted to understand the underlying neural mechanisms behind our findings.

As recognized, an effective information processing relies on three inseparable and interactive aspects, that is, attentional orienting, switching, and inhibition. Deficit in either aspect will lead to a damaged processing; for example, an individual with hyperactivity may fail to control himself from the interference of novel information and was found with deficit in attentional inhibition by the stop-signal task (Rasmussen et al., 2015; Grane et al., 2016). In this work, RP patients exhibited a deficit in the attentional orienting and flexibility but not in attentional inhibition. Although the stop-signal task applied in this study was structured with 53.8% GO trials and 46.2% STOP trials, it was shown that the accuracy increases with the difficulty of inhibition (SOA), and its accuracy curve is similar to that of the typical stop-signal task with a high proportion of GO trials (∼75%) (data not shown), indicating the validity of our experimental design. As such, the presented normal inhibition function in RP patients may, in turn, provide additional evidence that the impairment in spatial processing should be attributable to the deficit in attentional orienting and flexibility revealed in this study.

Additional information about recognition processing is obtained from the EZ-diffusion model analysis. Generally, an increase in RT could be interpreted by a slow motor response (Ter), increased boundary separation (A), and/or decreased drift rate (*v*) (Ratcliff et al., 2016). Notably, the result of Experiment 4 gave the evidence for the impulsive information processing style (i.e., significantly lower in boundary estimates). Therefore, it is quite likely that the slow performance of RP patients was associated with either the reduction in drift rate, or the increase in non-decision time, or both. Since the drift rate represents the rate of information accumulation and reflects the efficiency of information processing (Karalunas et al., 2012), its reduction found in Experiments 1, 2, and 4 indicates a general impairment in the information processing in RP patients, leading to reduced reaction speed. Similar to patients with visual field loss including glaucoma and unilateral anterior ischemic optic neuropathy, RP patients exhibited longer RT than their healthy counterparts in certain experiments (Nowomiejska et al., 2010). Additionally, RP exerts an increase in the non-decision time in these three experiments. Considering that all responses were key responses in these experiments, non-decision time mainly reflects the duration of information encoding. The information encoding may link to the visual acuity in some way. It is speculated that impaired visual acuity or restricted visual field may credit to reduced fixation stability for amblyopic and RP patients (Chung et al., 2015; Zipori et al., 2018; Raveendran et al., 2019), suggesting that the poor information encoding could be caused by the visual instability in RP patients. Further study on RP patients with poor visual acuity demonstrated that eye-movement training may lead to an improvement in the recognition performance (Yoshida et al., 2014).

Interestingly, the RP-induced extension during the encoding processing in attentional orienting and switching could partly give the reason for the slow performance of RP patients presented in Experiments 1 and 4. Also, V1 area may be involved in the impairment during the information processing as observed from RP patients. This abnormal activation of V1 area due to the reduction in pigment optical density of cone photoreceptors was related to the information processing, including the spatial perception, and discrimination, attentional shifting (Chirimuuta et al., 2003; Fortenbaugh et al., 2008; Eichhorn et al., 2009). On the other hand, the deficit in boundary separation was only found in the attentional switching task, suggesting a possible impairment in the information processing caution in RP patients.

## Conclusion

Or study found that RP exerted impairment in spatial processing mainly in the peripheral visual field, which may be attributable to the decrease of information processing speed and increase of information encoding time. Moreover, specific to the peripheral visual field, RP patients exhibited normal inhibition function but impaired attentional orienting and flexibility. The impairment of attentional orienting is mainly related to the decrease of processing speed and poor performance of information encoding. Meanwhile, the impaired attentional flexibility is quite likely related to the prolongation of information encoding time under sustained condition due to visual instability.

## Data Availability Statement

The raw data supporting the conclusions of this article will be made available by the authors, without undue reservation.

## Ethics Statement

The studies involving human participants were reviewed and approved by the Ethics Committee of Capital Medical University. The patients/participants provided their written informed consent to participate in this study.

## Author Contributions

Y-LL, J-FL, and G-XN conceived the study, participated in the design, and wrote most of the manuscript. S-FZ, Y-YW, LZ, Y-LY, M-WG, and C-QL performed the experiments. Y-YW and Y-LY analyzed the data and helped in drafting the manuscript. All authors read and approved the final manuscript.

## Conflict of Interest

The authors declare that the research was conducted in the absence of any commercial or financial relationships that could be construed as a potential conflict of interest.

## References

[B1] Akeo K. Hiida Y. Saga M. Inoue R. Oguchi Y. (2002). Correlation between contrast sensitivity and visual acuity in retinitis pigmentosa patients. *Ophthalmologica* 216 185–191. 10.1159/000059627 12065855

[B2] Alexander K. R. Derlacki D. J. Fishman G. A. (1992a). Contrast thresholds for letter identification in retinitis pigmentosa. *Invest. Ophthalmol. Vis. Sci.* 33 1846–1852. 1582787

[B3] Alexander K. R. Derlacki D. J. Fishman G. A. (1995). Visual acuity versus letter contrast sensitivity in retinitis pigmentosa. *Vision Res.* 35 1495–1499. 10.1016/s0042-6989(97)00382-9 7645278

[B4] Alexander K. R. Derlacki D. J. Fishman G. A. (1999). Coherence and the judgment of spatial displacements in retinitis pigmentosa. *Vision Res.* 39 2267–2274. 10.1016/s0042-6989(98)00320-4 10343808

[B5] Alexander K. R. Derlacki D. J. Fishman G. A. Peachey N. S. (1991). Acuity-luminance and foveal increment threshold functions in retinitis pigmentosa. *Invest Ophthalmol. Vis. Sci.* 32 1446–1454. 2016127

[B6] Alexander K. R. Derlacki D. J. Fishman G. A. Szlyk J. P. (1992b). Grating, vernier, and letter acuityin retinitis pigmentosa. *Invest Ophthalmol. Vis. Sci.* 33 3400–3406. 1428713

[B7] Alexander K. R. Derlacki D. J. Xie W. Fishman G. A. Szlyk J. P. (1998). Discrimination of spatial displacements by patients with retinitis pigmentosa. *Vision Res.* 38 1171–1181. 10.1016/S0042-6989(97)00235-6 9666975

[B8] Alexander K. R. Hutman L. P. Fishman G. A. (1986). Dark-adapted foveal thresholds and visual acuity in retinitis pigmentosa. *Arch. Ophthalmol.* 104 390–394. 10.1001/archopht.1986.01050150090034 3954640

[B9] Baranton K. Nguyen T. H. Yoshida M. Giraudet G. (2014). Comparing V1 between myopes and emmetropes. *J. Vision* 14 686–686. 10.1167/14.10.686

[B10] Chen C. Zhang X. Wang Y. Zhou T. Fang F. (2016). Neural activities in V1 create the bottom-up saliency map of natural scenes. *Exp. Brain Res.* 234 1769–1780. 10.1007/s00221-016-4583-y 26879771

[B11] Chirimuuta M. Clatworthy P. L. Tolhurst D. J. (2003). Coding of the contrasts in natural images by visual cortex (V1) neurons: a bayesian approach. *J. Opt. Soc. Am. A Opt. Image Sci. Vis.* 20 1253–1260. 10.1364/josaa.20.001253 12868631

[B12] Chung S. T. Kumar G. Li R. W. Levi D. M. (2015). Characteristics of fixational eye movements in amblyopia: limitationson fixation stability and acuity? *Vision Res.* 114 87–99. 10.1016/j.visres.2015.01.016 25668775 PMC4529398

[B13] Eichhorn J. Sinz F. Bethge M. (2009). Natural image coding in V1: how much use is orientation selectivity? *PLoS Comput. Biol.* 5:e1000336. 10.1371/journal.pcbi.1000336 19343216 PMC2658886

[B14] Farrant K. Uddin L. Q. (2015). Asymmetric development of dorsal and ventral attention networks in the human brain. *Dev. Cogn. Neurosci.* 12 165–174. 10.1016/j.dcn.2015.02.001 25797238 PMC4396619

[B15] Ferreira S. Pereira A. C. Quendera B. Reis A. Silva E. D. Castelobranco M. (2017). Primary visual cortical remapping in patients with inherited peripheral retinal degeneration. *Neuroimage Clin.* 13 428–438. 10.1016/j.nicl.2016.12.013 28116235 PMC5233796

[B16] Fortenbaugh F. C. Hicks J. C. Turano K. A. (2008). The effect of peripheral visual field loss on representations of space: evidence for distortion and adaptation. *Invest Ophthalmol. Vis. Sci.* 49:2765. 10.1167/iovs.07-1021 18515599

[B17] Gordon I. E. Johns E. (1984). A visual aid for artists and others with retinitis pigmentosa (‘Tunnel Vision’). *Leonardo* 17 202–204. 10.2307/1575192

[B18] Grane V. A. Brunner J. F. Endestad T. Aasen I. E. Kropotov J. Knight R. T. (2016). ERP correlates of proactive and reactive cognitive control in treatment-naïve adult ADHD. *PLoS One* 11:e0159833. 10.1371/journal.pone.0159833 27448275 PMC4957760

[B19] Hamel C. (2006). Retinitis pigmentosa. *Orphanet J. Rare Dis.* 1:40. 10.1186/1750-1172-1-40 17032466 PMC1621055

[B20] Herse P. (2005). Retinitis pigmentosa: visual function and multidisciplinary management. *Clin. Exp. Optom.* 88 335–350. 10.1111/j.1444-0938.2005.tb06717.x 16255692

[B21] Karalunas S. L. Huang-pollock C. L. Nigg J. T. (2012). Decomposing ADHD-related effects in response speed and variability. *Neuropsychology* 26 684–694. 10.1037/a0029936 23106115 PMC3516369

[B22] Kerns J. G. Cohen J. D. MacDonald A. W. Cho R. Y. Stenqer V. A. Carter C. S. (2004). Anterior cingulate conflict monitoring and adjustments in control. *Science* 303 1023–1029. 10.1126/science.1089910 14963333

[B23] Li Z. (1999). Contextual influences in V1 as a basis for pop out and asymmetry in visual search. *Proc. Natl. Acad. Sci. U.S.A.* 96 10530–10535. 10.1073/pnas.96.18.10530 10468643 PMC17923

[B24] Marmor M. F. (1981). Contrast sensitivity and retinal disease. *Ann. Ophthalmol.* 13 1069–1071. 7340659

[B25] Mayr U. Keele S. W. (2000). Changing internal constraints on action: the role of backward inhibition. *J. Exp. Psychol. Gen.* 129 4–26. 10.1037/0096-3445.129.1.4 10756484

[B26] Meiran N. Chorev Z. (2005). Phasic alertness and the residual task-switching cost. *Exp. Psychol.* 52:109. 10.1027/1618-3169.52.2.109 15850158

[B27] Metin B. Roeyers H. Wiersema J. R. van der Meere J. J. Thompson M. Sonugabarke E. (2013). ADHD performance reflects inefficient but not impulsive information processing: a diffusion model analysis. *Neuropsychology* 27 193–200. 10.1037/a0031533 23527647

[B28] Monsell S. (2003). Task switching. *Trends Cogn. Sci.* 7 134–140. 10.1016/S1364-6613(03)00028-7 12639695

[B29] Mori T. Matsuura K. Zhang B. Smith E. L. Chino Y. M. (2002). Effects of the duration of early strabismus on the binocular responses of neurons in the monkey visual cortex (V1). *Invest Ophthalmol. Vis. Sci.* 43 1262–1269. 10.1007/s00417-002-0449-z 11923274

[B30] Moustafa A. A. Kéri S. Somlai Z. Balsdon T. Frydecha D. Misiak B. (2015). Drift diffusion model of reward and punishment learning in schizophrenia: modeling and experimental data. *Behav. Brain Res.* 291 147–154. 10.1016/j.bbr.2015.05.024 26005124

[B31] Nowomiejska K. Vonthein R. Paetzold J. Zagorski Z. Kardon R. Schiefer U. (2010). Reaction time during semi-automated kinetic perimetry (skp) in patients with advanced visual field loss. *Acta Ophthalmol.* 88 65–69. 10.1111/j.1755-3768.2008.01407.x 19094165

[B32] Rasmussen J. Casey B. J. van Erp T. G. Tamm L. Epstein J. N. Buss C. (2015). ADHD and cannabis use in young adults examined using fMRI of a go/nogo task. *Brain Imaging Behav.* 10 1–11. 10.1007/s11682-015-9438-9 26489976 PMC4840078

[B33] Ratcliff R. (1978). A theory of memory retrieval. *Psychol. Rev.* 85 59–108. 10.1037/0033-295X.85.2.59

[B34] Ratcliff R. Smith P. L. Brown S. D. Mckoon G. (2016). Diffusion decision model: current issues and history. *Trends Cogn. Sci.* 20 260–281. 10.1016/j.tics.2016.01.007 26952739 PMC4928591

[B35] Raveendran R. N. Bobier W. Thompson B. (2019). Reduced amblyopic eye fixation stability cannot be simulated using retinal-defocus-induced in visual acuity. *Vision Res.* 154 14–20. 10.1016/j.visres.2018.10.005 30389388

[B36] Sandberg M. A. Berson E. L. (1983). Visual acuityand cone spatial density in retinitispigmentosa. *Invest Ophthalmol. Vis. Sci.* 24 1511–1513. 6642929

[B37] Silver M. Ress D. D. (2007). Neural correlates of sustained spatial attention in human early visual cortex. *J. Neurophysiol.* 97 229–237. 10.1152/jn.00677.2006 16971677 PMC1868502

[B38] Szlyk J. P. Seiple W. Xie W. (1995). Symmetry discrimination in patients with retinitis pigmentosa. *Vision Res.* 35 1633–1640. 10.1016/0042-6989(94)00275-q 7667920

[B39] Temme L. A. Maino J. H. Noell W. K. (1985). Eccentricity perception in the periphery of normal observers and those with retinitis pigmentosa. *Am. J. Optom. Physiol. Opt.* 62 736–743. 10.1097/00006324-198511000-00003 4073209

[B40] Thakral P. P. Slotnick S. D. (2009). The role of parietal cortex during sustained visual spatial attention. *Brain Res.* 1302 157–166. 10.1016/j.brainres.2009.09.031 19765554

[B41] Thiel A. Sireteanu R. (2009). Strabismic amblyopes show a bilateral rightward bias in a line bisection task: evidence for a visual attention deficit. *Vision Res.* 49 287–294. 10.1016/j.visres.2008.08.005 18775742

[B42] Turano K. (1991). Bisection judgements in patients with retinitis pigmentosa. *Clin. Vis. Sci.* 6 119–130.

[B43] Turano K. Herdman S. J. Dagnelie G. (1993). Visual stabilization of posture in retinitis pigmentosa and in artificially restricted visual fields. *Invest Ophthalmol. Vis. Sci.* 34 3004–3010. 8360031

[B44] Turano K. Wang X. (1992). Motion thresholds inretinitis pigmentosa. *Invest Ophthalmol. Vis. Sci.* 33 2411–2422. 1634338

[B45] Turatto M. Facoetti A. Serra G. Benso F. Angi M. Umiltà C. (1999). Visuospatial attention in myopia. *Brain Res. Cogn. Brain Res.* 8 369–372. 10.1016/S0926-6410(99)00025-7 10556613

[B46] Vincent J. L. Kahn I. Snyder A. Z. Raichle M. E. Buckner R. L. (2008). Evidence for a frontoparietal control system revealed by intrinsic functional connectivity. *J. Neurophysiol.* 100 3328–3342. 10.1152/jn.90355.2008 18799601 PMC2604839

[B47] Wagenmakers E. J. van der Maas H. L. Grasman R. P. (2017). An EZ-diffusion model for response time and accuracy. *Psychon. Bull. Rev.* 14 3–22. 10.3758/bf03194023 17546727

[B48] Wilkins A. J. Shallice T. Mccarthy R. (1987). Frontal lesions and sustained attention. *Neuropsychologia* 25 359–365. 10.1016/0028-3932(87)90024-8 3601041

[B49] Wittich W. Faubert J. Watanabe D. H. Kapusta M. A. Overbury O. (2011). Spatial judgments in patients with retinitis pigmentosa. *Vision Res.* 51 165–173. 10.1016/j.visres.2010.11.003 21073889

[B50] Yoshida M. Origuchi M. Urayama S. Takatsuki A. Kan S. Aso T. (2014). fMRI evidence of improved visual function in patients with progressive retinitis pigmentosa by eye-movement training. *Neuroimage Clin.* 5 161–168. 10.1016/j.nicl.2014.02.007 25068106 PMC4109700

[B51] Zipori A. B. Colpa L. Wong A. M. F. Cushing S. L. Gordon K. A. (2018). Postural stability and visual impairment:Assessing balance in children with strabismus and amblyopia. *PLoS One* 13:e0205857. 10.1371/journal.pone.0205857 30335817 PMC6193669

